# Associações entre Obesidade Eutrófica e Alterações no Perfil Lipídico de Adultos Jovens

**DOI:** 10.36660/abc.20220914

**Published:** 2023-10-06

**Authors:** Anna Flavia Ferreira Passos, Acsa de Castro Santos, Alexandre Siqueira Guedes Coelho, Cristiane Cominetti

**Affiliations:** 1 Grupo de Pesquisa em Genômica Nutricional Faculdade de Nutrição Universidade Federal de Goiás Goiânia GO Brasil Grupo de Pesquisa em Genômica Nutricional , Faculdade de Nutrição , Universidade Federal de Goiás , Goiânia , GO – Brasil; 2 Faculdade de Agronomia Universidade Federal de Goiás Goiânia GO Brasil Faculdade de Agronomia , Universidade Federal de Goiás , Goiânia , GO – Brasil

**Keywords:** Apolipoproteínas, Composição Corporal, Dislipidemias, Doenças Cardiovasculares, Glicemia

## Abstract

**Fundamento:**

A obesidade eutrófica (OE) é caracterizada por índice de massa corporal (IMC) normal, mas com alto percentual de gordura corporal (%GC), o que aumenta os riscos de comorbidades cardiometabólicas. A avaliação e interpretação precisas dos dados de composição corporal são necessárias para reduzir esses riscos.

**Objetivos:**

Comparar o perfil cardiometabólico de indivíduos com OE e %GC normal e avaliar os fatores de risco associados.

**Métodos:**

Foi realizado estudo transversal com 222 adultos brasileiros de uma comunidade universitária, dos quais 157 tinham OE e 65 tinham IMC e %GC normais (grupo sem OE). Todos os participantes relataram ser assintomáticos e sem problemas de saúde subjacentes. Foram avaliadas características socioeconômicas, estilo de vida, consumo alimentar, antropometria, medidas de composição corporal (por meio de absorciometria radiológica de dupla energia) e perfis lipídico e glicêmico. Valor de p < 0,05 foi estabelecido como significativo.

**Resultados:**

A mediana de idade dos participantes foi de 23 anos (intervalo interquartil: 21 a 25), sendo a maioria do sexo feminino (67,1%). Não foram encontradas diferenças significativas na pressão arterial, idade ou nível de atividade física entre os grupos com e sem OE. No entanto, a frequência de distúrbios do perfil lipídico foi maior no grupo com OE (54%) em comparação com o grupo sem OE (34%) (p < 0,006). Circunferência do pescoço, %GC e distúrbios do perfil lipídico foram positivamente associados com a OE.

**Conclusão:**

Indivíduos com OE apresentam pior perfil cardiometabólico do que aqueles sem OE, e essa condição está associada a importantes biomarcadores. Torna-se importante abordar esses resultados para prevenir complicações cardiometabólicas de longo prazo. A avaliação e a interpretação precisas dos dados da composição corporal, independentemente do IMC, são cruciais nesse cenário.

## Figura Central

**Figure:**
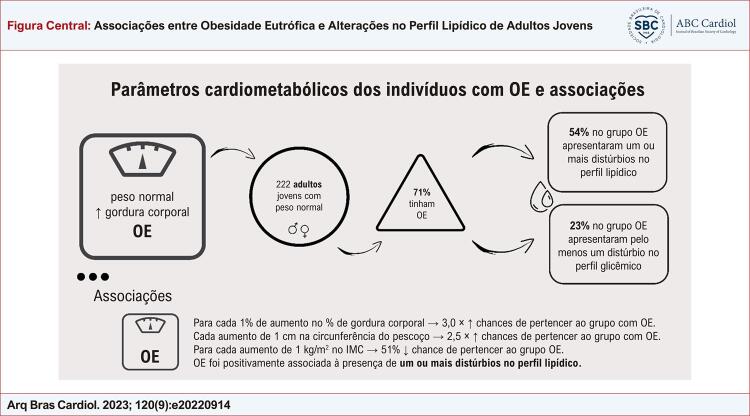
: Associações entre Obesidade Eutrófica e Alterações no Perfil Lipídico de Adultos Jovens

**Figure:** : Associações entre Obesidade Eutrófica e Alterações no Perfil Lipídico de Adultos Jovens

IMC: índice de massa corporal; OE: obesidade eutrófica.

## Introdução

A obesidade eutrófica (OE), descrita em 2006, é uma condição caracterizada por índice de massa corporal (IMC) normal e alto percentual de gordura corporal (%GC). ^1^ Foi descrita em razão de falhas nos conceitos de IMC e obesidade. A obesidade é definida pela Organização Mundial da Saúde como excesso de gordura corporal associada a riscos à saúde ^1 , 2^ e é diagnosticada com base no IMC. No entanto, o IMC é conhecido por ser um índice falho, especialmente quando aplicado em nível individual, pois não diferencia massa corporal magra de massa corporal gorda. ^2 , 3^

Como resultado do excesso de %GC, indivíduos com OE apresentam maior risco de desenvolver síndrome metabólica, dislipidemia aterogênica, obesidade e doenças cardiometabólicas, incluindo diabetes mellitus tipo 2 e doenças cardiovasculares (DCV), em comparação com indivíduos com %GC normal. ^1 , 3 - 5^ As DCV são a principal causa de morte em todo o mundo, e existe uma projeção de que serão responsáveis por mais de 23 milhões de mortes até 2030. ^6^

Portanto, considerando a importância da adiposidade como fator de risco para DCV e outras doenças crônicas não transmissíveis relacionadas à nutrição, a avaliação do %GC em indivíduos com IMC normal é fundamental para diagnóstico preciso e intervenções precoces. ^7 , 8^ Estima-se que aproximadamente 30 milhões de pessoas nos Estados Unidos apresentam OE, ^9^ e alguns estudos brasileiros sobre esse assunto ^4 , 10 , 11^ revelaram que a OE está associada à síndrome metabólica e à resistência à insulina (RI). ^4^ Em estudo prévio de nosso grupo, observamos alta frequência de dislipidemia em indivíduos com OE. ^10^ Esses resultados reforçam a relação entre OE e distúrbios nos biomarcadores lipídicos e glicêmicos, que são importantes preditores de DCV e desenvolvimento de diabetes mellitus tipo 2.

Com base na hipótese de que o %GC elevado pode resultar em alterações no perfil cardiometabólico, mesmo na presença de IMC normal e, pela importância de compreender os aspectos metabólicos da OE, o presente estudo visou comparar o perfil cardiometabólico de indivíduos com e sem OE, bem como avaliar suas associações com dados socioeconômicos, antropométricos, de composição corporal, bioquímicos e de consumo alimentar.

## Métodos

### Desenho do estudo e participantes

**Figura 1:**
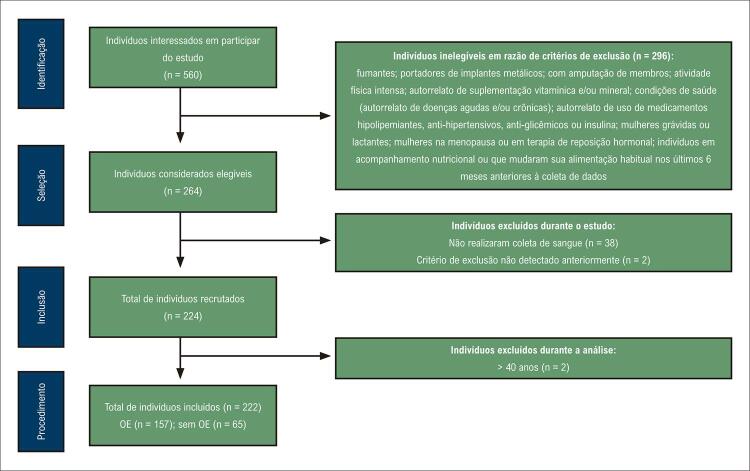
– Fluxograma de recrutamento de participantes IMC: índice de massa corporal; OE: obesidade eutrófica.

Trata-se de um estudo transversal com recrutamento e coleta de dados no período de janeiro a junho de 2019. O estudo foi divulgado por meio de folders, redes sociais e e-mails enviados a alunos, professores e demais funcionários da Universidade Federal de Goiás. Foram incluídos indivíduos que apresentaram IMC normal (entre 18,50 e 24,99 kg/m ^2^ ) ^2^ e idade entre 20 e 59 anos. Conforme descrito na Figura 1 , não foram incluídos: fumantes; portadores de implantes metálicos ou com amputação de membros; indivíduos que praticavam atividade física intensa (atletas ou praticantes regulares de exercícios de alto rendimento); indivíduos que autorrelataram uso de suplementação vitamínica e/ou mineral, presença de doenças agudas e/ou crônicas, uso de medicamentos hipolipemiantes, anti-hipertensivos, antiglicêmicos ou insulina; mulheres grávidas ou lactantes; mulheres na menopausa ou em terapia de reposição hormonal; indivíduos em acompanhamento nutricional e/ou que mudaram sua alimentação habitual nos últimos 6 meses anteriores à coleta de dados; ou aqueles que perderam alguma etapa da coleta de dados. Esses critérios de exclusão foram implementados para gerenciar possíveis variáveis de confusão que podem introduzir viés na relação entre o excesso de gordura corporal e as variáveis investigadas neste estudo.

Os indivíduos foram agrupados por sexo e idade, e foram aplicados os pontos de corte utilizados em dois estudos bem estabelecidos sobre OE para classificar o %GC. Os critérios para pontos de corte foram selecionados com base na consistência do método de avaliação da composição corporal. Inicialmente, foi utilizada a referência dos autores que originalmente definiram a OE ^1^ em mulheres; no entanto, eles não forneceram pontos de corte para homens. Para abordar essa lacuna, buscou-se uma referência para indivíduos do sexo masculino com um ponto de corte mais sensível que ainda capturasse os fatores de risco associados. ^12^ Para mulheres e homens, pontos de corte > 30% ^1^ e > 19%, ^12^ respectivamente, foram aplicados para classificar o %GC como elevado. Considerando a falta de um critério padronizado para classificar a prevalência de OE, adotamos um conjunto de limiares propostos para sobrepeso e obesidade em adultos, considerando a associação entre o excesso de %GC e riscos à saúde. ^1 , 2^ Os limites propostos para classificação foram determinados da maneira seguinte: < 20% para muito baixo, 20% a < 30% para baixo, 30% a < 50% para moderado, 50% a < 70% para alto e ≥ 70% para muito alto. ^13^

### Coleta de dados

Os dados foram coletados na Faculdade de Nutrição e na Unidade de Pesquisa Clínica do Hospital das Clínicas da Universidade Federal de Goiás. Primeiramente, os participantes receberam informações sobre o estudo e assinaram um termo de consentimento livre e esclarecido. Foi aplicado um questionário para avaliar dados socioeconômicos, demográficos, de saúde, estilo de vida e consumo alimentar (recordatório alimentar de 24 horas). Foram realizadas 3 medidas não consecutivas de pressão arterial, ^14^ absorciometria radiológica de dupla energia (DXA) e avaliação antropométrica. ^15^ Também foram determinados a classificação econômica ^16^ e o nível de atividade física. ^17^

Amostras de sangue foram coletadas da veia cubital mediana após jejum de 12 horas por um profissional habilitado. Imediatamente após a coleta, as amostras de sangue foram transferidas para tubos apropriados para obtenção de soro e/ou plasma e, em seguida, enviadas ao laboratório para análises bioquímicas. Os indivíduos foram orientados a não consumir bebidas alcoólicas ou praticar atividade física intensa nas 72 horas anteriores à coleta de sangue. Foram também instruídos a manter sua dieta habitual e um peso estável nas últimas 2 semanas anteriores à coleta de sangue. ^18 - 20^ Dois outros recordatórios alimentares de 24 horas não consecutivos, incluindo um dia de fim de semana, foram coletados. ^21^

### Antropometria e composição corporal

A massa corporal foi medida em uma balança digital Filizola ^®^ (Filizola Shop, São Paulo, Brasil) e a estatura foi determinada com um estadiômetro Seca ^®^ (Seca Deutschland, Hamburgo, Alemanha). ^15^ As circunferências da cintura e do pescoço foram medidas em duplicata com uma fita métrica corporal Seca ^®^ com 200 cm de comprimento e precisão de 1 mm (Seca Deutschland, Hamburgo, Alemanha), e foi usado o valor médio para análise dos dados. A composição corporal foi medida usando um dispositivo DPX NT Lunar ^®^ DXA (General Electric Medical Systems, Madison, EUA). ^22^

### Biomarcadores cardiometabólicos

Foram avaliadas as concentrações sanguíneas de colesterol total (CT), triacilglicerol (TG), colesterol em lipoproteínas de baixa densidade (LDL-c), colesterol em lipoproteínas de alta densidade (HDL-c), colesterol em lipoproteínas de muito baixa densidade (VLDL-c), apolipoproteína (Apo) A1, Apo B, glicose, insulina e hemoglobina glicada (HbA1c). Os biomarcadores do perfil lipídico sérico foram determinados a partir do método enzimático colorimétrico direto. As concentrações de LDL-c, VLDL-c e não HDL-c foram estimadas por meio de equações. ^19 , 21^ Foram usados os seguintes pontos de corte para classificar alterações dos marcadores: CT ≥ 190, LDL-c ≥ 130, não HDL-c ≥ 160, TG ≥ 150, VLDL-c ≥ 30, HDL-c < 40 (todos em mg/dL); relação CT/HDL-c ≥ 4,4 para mulheres e ≥ 5,1 para homens; e razão LDL-c/HDL-c ≥ 2,9 para mulheres e ≥ 3,3 para homens. ^19 , 23 - 25^ As concentrações de Apo A1 e Apo B foram analisadas pelo método de turbidimetria e foram consideradas alteradas quando Apo A1 estava < 140 mg/dL para mulheres e < 120 mg/dL para homens ^22^ e quando Apo B estava ≥ 104 mg/dL para mulheres e homens. ^26^ A relação Apo B/Apo A1 foi considerada elevada quando estava ≥ 0,6 para mulheres e ≥ 0,7 para homens. ^27 , 28^ O índice aterogênico foi estimado, e os valores foram considerados altos quando > 2,24. ^29^

As concentrações séricas de glicose foram determinadas pelo método colorimétrico enzimático, adotando-se os valores de referência estabelecidos pela Sociedade Brasileira de Diabetes. ^20^ As concentrações sanguíneas totais de HbA1c foram avaliadas pelo método de inibição imunoturbidimétrica, aplicando-se os valores de referência da Sociedade Brasileira de Diabetes. ^20^ As concentrações séricas de insulina foram avaliadas por eletroquimioluminescência. Os índices *homeostasis model assessment of insulin resistance* (HOMA-IR), HOMA2-IR, HOMA *of beta-cell function* (HOMA-beta) e índice quantitativo de verificação da sensibilidade à insulina (QUICKI) foram calculados. ^20 , 30^ Os pontos de corte foram > 2,71 para HOMA-IR, > 1,80 para HOMA2-IR, acima do percentil 90 da amostra para HOMA-beta e inferior ao percentil 10 da amostra para o índice QUICKI. ^20 , 30^ O índice triacilglicerol-glicose (TyG) foi estimado, e foram usados como pontos de corte valores superiores a 4,55 para mulheres e 4,68 para homens. ^20^

### Consumo alimentar

Três recordatórios de 24 horas foram aplicados em dias não consecutivos, incluindo um dia de final de semana, ^20^ seguindo o método de passagens múltiplas. ^31^ Dois nutricionistas treinados aplicaram um recordatório de 24 horas presencialmente e dois recordatórios de 24 horas por meio de telefonemas. Para auxiliar na quantificação das porções de alimentos, foram utilizados um manual fotográfico e utensílios de medidas caseiras. ^32^ A conversão das medidas caseiras em gramas ^33^ e o gerenciamento dos dados foram padronizados. Os recordatórios alimentares de 24 horas foram avaliados por meio do software Nutrition Data System for Research software (NDSR, Nutrition Coordinating Center, University of Minnesota, Minneapolis–Saint Paul, EUA). ^34^ Foram aplicados os valores propostos pela Sociedade Brasileira de Cardiologia para macronutrientes, fibra alimentar e adequação de ácidos graxos. ^19^

### Análise estatística e justificativa do tamanho da amostra

Bancos de dados em dupla entrada foram construídos para verificar a consistência. Apesar de os critérios de seleção para a faixa etária adulta de 20 a 59 anos, com o recrutamento de apenas dois indivíduos com mais de 40 anos, estes foram excluídos das análises, permanecendo apenas adultos jovens com 39 anos ou menos, de modo que, em termos de idade, a amostra não apresentou esses valores atípicos. Considerando que não há estudos sobre OE com amostras representativas do Brasil, o cálculo amostral foi baseado no tamanho do efeito de Cohen para amostras independentes. ^35^ O poder estatístico para rejeitar a hipótese nula foi estabelecido em 80%, com probabilidade de erro tipo I de 0,05, e tamanhos de amostra de 157 e 65 observações para indivíduos com e sem OE, respectivamente. O tamanho do efeito estimado foi de 0,41, o que é considerado um valor médio. ^35^

A distribuição dos dados foi avaliada por meio do teste de Shapiro-Wilk. Teste t de Student não pareado ou teste de Mann-Whitney foram aplicados para comparação de médias, com base na distribuição dos dados. A análise de dados categóricos e da frequência de alterações no perfil cardiometabólico de indivíduos com e sem OE foi realizada por meio do teste χ ^2^ de Pearson ou do teste exato de Fisher. Os dados de ingestão de nutrientes foram ajustados para energia quando necessário. ^36^ As variáveis categóricas foram apresentadas como frequências (%) absolutas e relativas e as variáveis contínuas, como média ± desvio padrão ou mediana (intervalo interquartil) de acordo com a distribuição dos dados.

Utilizamos modelos de regressão logística múltipla para investigar as relações entre variáveis independentes e dependentes. Para aprimorar os modelos, usamos a estratégia *stepwise* , um procedimento automatizado para selecionar as variáveis preditoras mais significativas para incluir no modelo. As variáveis independentes foram sexo, cor da pele, idade, nível de atividade física, pressões arteriais sistólica e diastólica, peso, estatura, IMC, circunferências do pescoço e da cintura, %GC, %GC ginoide e androide, relação %GC androide/ginoide, glicemia de jejum e insulina, HOMA-IR, HOMA-2IR, HOMA-beta, HbA1c, índice QUICKI, Apo A1, Apo B, CT, HDL-c, LDL-c, não HDL-c, VLDL-c, TG, as relações CT/HDL-c, LDL-c/HDL-c e Apo B/Apo A1, TyG, índice aterogênico, presença de distúrbios nos perfis glicêmico e lipídico, energia, gordura total, carboidratos, proteínas, colesterol dietético, ácidos graxos saturados, mono- e poli-insaturados, e fibras alimentares. Todas as variáveis foram escolhidas de acordo com sua importância clínica com a condição estudada. P < 0,05 foi considerado estatisticamente significativo e todas as análises foram realizadas usando o software R versão 4.0.3. ^37^

## Resultados

Foram recrutados 222 indivíduos ( Figura 1 ), dos quais 67% eram mulheres. Os participantes do estudo tinham idade entre 20 e 34 anos e IMC normal. Seguindo a classificação de %GC, 157 (71%) participantes foram designados para o grupo OE, indicando alta prevalência, enquanto os 65 participantes restantes foram designados para o grupo sem OE. Considerando o sexo, 72,5% das mulheres e 67,1% dos homens apresentaram %GC aumentado. Em relação ao ponto de corte para classificação do %GC, utilizamos um valor > 30% ^1^ para classificar as mulheres como tendo OE. Contudo, visto que este ponto de corte pode ser questionado, examinamos as implicações de utilizar um ponto de corte superior a 32% ^12^ e observamos que 14 mulheres não preencheriam os critérios para a classificação em OE. Como resultado, o número total de indivíduos classificados como portadores de OE seria reduzido para 143, resultando em uma prevalência global de 64,4%, ainda considerada elevada. Porém, das 14 mulheres que não seriam classificadas como tendo OE, 11 apresentaram distúrbios nos perfis lipídico e glicêmico, indicando a presença de fatores de risco associados ao excesso de %GC.

A Tabela 1 descreve os dados socioeconômicos, de estilo de vida, antropométricos, de composição corporal e bioquímicos dos indivíduos dos grupos com e sem OE. Os indivíduos do grupo OE apresentaram maiores valores de peso, IMC, circunferência da cintura, %GC, %GC androide e ginoide, relação androide/ginoide, insulina, HOMA-IR, HOMA-beta, TyG, CT, LDL-c, não HDL-c, VLDL-c, TG, relação CT/HDL-c, relação LDL-c/HDL-c, Apo B, relação Apo B/Apo A1, bem como valores mais baixos do índice QUICKI do que aqueles no grupo sem OE.

**Tabela 1:** – Variáveis socioeconômicas, de estilo de vida, antropométricas, de composição corporal e bioquímicas da amostra total e dos grupos com e sem OE

Variáveis	Total (n = 222, 100.0%)	OE (n = 157, 70.7%)	Sem OE (n = 65, 29.3%)	Valor p
**Sexo**				0,410
Masculino	73 (32,9)	49 (31,2)	24 (36,9)	
Feminino	149 (67,1)	108 (68,8)	41 (63,1)	
**Cor da pele**				0,522
Branca	84 (37,8)	64 (40,8)	20 (30,8)	
Parda	94 (42,3)	63 (40,1)	31 (47,7)	
Preta	29 (13,1)	19 (12,1)	10 (15,4)	
Amarela	15 (6,8)	11 (7,0)	4 (6,2)	
**Estado civil**				0,522
Solteiro	199 (89,6)	138 (87,9)	61 (93,8)	
Casado	22 (9,9)	18 (11,5)	4 (6,2)	
Divorciado	1 (0,5)	1 (0,6)	0 (0)	
**Nível de escolaridade**				0,065
Superior incompleto	167 (75,2)	114 (72,6)	53 (81,5)	
Superior completo	44 (19,8)	32 (20,4)	12 (18,5)	
Pós-graduação completa	11 (5,0)	11 (7,0)	0 (0)	
**Classe socioeconômica**				0,094
Alta	143 (64,4)	106 (67,5)	37 (56,9)	
Intermediária	78 (35,1)	51 (32,5)	27 (41,5)	
Baixa	1 (0,5)	0 (0)	1 (1,5)	
Idade (anos)	23,0 (21,0 – 25,0)	23,0 (21,0 – 26,0)	23,0 (21,0 – 24,0)	0,506
PAS (mmHg)	109,5 (100,0 – 115,8)	109,5 (100,0 – 116,0)	110,0 (100,0 – 115,0)	0,682
PAD (mmHg)	67,0 (60,1 – 74,0)	66,5 (60,0 – 74,0)	70,0 (62,0 – 74,0)	0,349
Peso (kg)	60,6 (55,0 – 66,6)	61,3 (56,0 – 66,7)	57,4 (53,0– 65,9)	0,026
Estatura (m)	1,7 (1,6 – 1,7)	1,7 (1,6 – 1,7)	1,7 (1,6 – 1,7)	0,719
IMC (kg/m ^2^ )	21,8 (20,5 – 23,0)	22,1 (21,0 – 23,3)	20,8 (19,8 – 22,0)	<0,0001
Nível de atividade física (MET min/semana)	260,0 (131,2 – 409,8)	240,0 (100,0 – 415,0)	280 (180,0 – 409,0)	0,210
CP (cm)	32,4 (31,2 – 36,0)	32,5 (31,4 – 35,8)	32,1 (30,7 – 36,1)	0,490
CC (cm)	72,7 ± 5,5	73,8 ± 5,2	70,1 ± 5,4	<0,0001
%GC DXA	30,4 (23,4 – 36,4)	34,2 (28,8 – 37,7)	24,6 (15,4 – 28,1)	<0,0001
%GC androide	30,7 ± 9,6	34,7 ± 7,6	20,9 ± 6,4	<0,0001
%GC ginoide	42,8 (34,2 – 48,4)	46,5 (38,1 – 49,5)	36,8 (23,2 – 41,0)	<0,0001
A/G	0,8 (0,7 – 0,9)	0,8 (0,7 – 0,9)	0,6 (0,6 – 0,8)	<0,0001
Glicose (mg/dL)	85,0 (80,2 – 89,8)	85,0 (81,0 – 90,0)	84,0 (80,0 – 89,0)	0,392
Insulina (pmol/L)	49,9 (38,9 – 66,6)	54,5 (42,4 – 71,8)	43,0 (35,2 – 54,5)	0,0001
HOMA-IR	1,4 (1,1 – 2,0)	1,6 (1,2 – 2,1)	1,2 (1,0 – 1,6)	0,0001
HOMA2-IR	0,9 (0,7 – 1,2)	1,0 (0,8 – 1,3)	0,8 (0,6 – 1,0)	0,0001
HOMA-beta	122,0 (87,9 – 170,1)	134,0 (95,1 – 176,1)	104,5 (78,9 – 149,6)	0,011
HbA1c%	4,8 (4,6 – 5,0)	4,8 (4,6 – 5,0)	4,8 (4,6 – 5,0)	0,953
Índice QUICKI	0,36 ± 0,02	0,36 ± 0,02	0,37 ± 0,02	<0,0001
CT (mg/dL)	175,7 ± 32,0	179,8 ± 33,1	165,8 ± 26,9	0,002
HDL-c (mg/dL)	55,0 (47,0 – 65,0)	55,0 (47,0 – 65,0)	56,0 (48,0 – 65,0)	0,782
LDL-c (mg/dL)	99,0 (82,0 – 117,8)	103,0 (83,0 – 118,0)	93,0 (74,0 – 107,0)	0,008
Não HDL-c (mg/dL)	115,0 (96,2 – 136,0)	121,0 (100,0 – 140,0)	109,0 (90,0 – 121,0)	0,002
VLDL-c (mg/dL)	14,8 (10,6 – 20,8)	16,4 (11,4 – 21,8)	12,8 (9,6 – 16,6)	0,001
TG (mg/dL)	74,0 (53,2 – 104,0)	82,0 (57,0 – 109,0)	64,0 (48,0 – 83,0)	0,001
Relação CT/HDL-c	3,0 (2,6 – 3,6)	3,1 (2,7 – 3,8)	2,8 (2,5 – 3,3)	0,021
Relação LDL-c/HDL-c	1,8 (1,4 – 2,3)	1,8 (1,4 – 2,4)	1,7 (1,3 – 2,0)	0,050
Apo A1 (mg/dL)	151,0 (137,0 – 168,0)	151,5 (138,8 – 172,2)	149,0 (137,0 – 163,0)	0,246
Apo B (mg/dL)	76,0 (65,0 – 89,0)	80,4 ± 19,0	70,6 ± 14,8	0,0003
Relação Apo B/Apo A1	0,5 (0,4 – 0,6)	0,5 (0,4 – 0,6)	0,5 (0,4 – 0,5)	0,031
TyG	3,5 ± 0,2	3,5 ± 0,2	3,4 ± 0,2	0,001
Índice aterogênico	1,0 (0,7 – 1,5)	1,0 (0,8 – 1,7)	0,9 (0,6 – 1,2)	0,022

Os dados são apresentados como média ± desvio padrão, mediana (intervalo interquartil) ou frequências absolutas e relativas. A/G: relação entre percentual de gordura corporal androide e ginoide; Apo A1: apolipoproteína A1; Apo B: apolipoproteína B; CC: circunferência da cintura; CP: circunferência do pescoço; CT: colesterol total; HbA1c: hemoglobina glicada; HDL-c: colesterol em lipoproteínas de alta densidade; HOMA-beta: modelo de avaliação da homeostase da função das células beta; HOMA-IR: modelo de avaliação da homeostase da resistência à insulina; HOMA2-IR: modelo de avaliação da homeostase 2 da resistência à insulina; IMC: índice de massa corporal; LDL-c: colesterol em lipoproteínas de baixa densidade; MET: equivalentes metabólicos; não HDL-c: colesterol não-HDL; PAD: pressão arterial diastólica; PAS: pressão arterial sistólica; %GC androide: percentual de gordura corporal androide; %GC DXA: percentual de gordura corporal avaliado por absorciometria radiológica de dupla energia; %GC ginoide: percentual de gordura corporal ginoide; OE: obesidade eutrófica; TG: triacilglicerol; TyG: índice de triacilglicerol-glicose; VLDL-c: colesterol em lipoproteínas de muito baixa densidade. Diferenças significativas entre os grupos: teste t de Student ou teste de Mann-Whitney, teste χ ^2^ de Pearson ou teste exato de Fisher.

**Figura 2:**
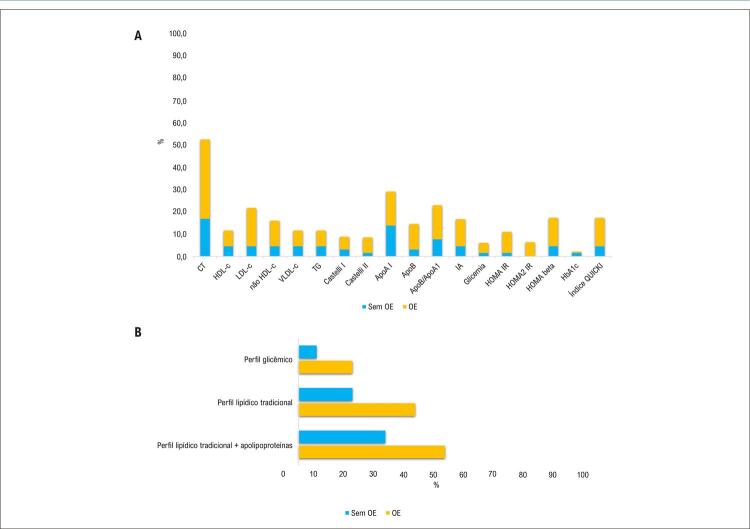
– A) Frequência de distúrbios do perfil lipídico e glicêmico nos grupos com e sem obesidade eutrófica. B) Frequência de pelo menos um distúrbio do perfil lipídico ou glicêmico nos grupos com e sem obesidade eutrófica. Apo A1: apolipoproteína A1; Apo B: apolipoproteína B; CT: colesterol total; HbA1c: hemoglobina glicada; HDL-c: colesterol em lipoproteínas de alta densidade; HOMA-beta: modelo de avaliação da homeostase da função das células beta; HOMA-IR: modelo de avaliação da homeostase da resistência à insulina; HOMA2-IR: modelo de avaliação da homeostase 2 da resistência à insulina; IA: índice aterogênico; LDL-c: colesterol em lipoproteínas de baixa densidade; não HDL-c: colesterol não-HDL; OE: síndrome de obesidade eutrófica; TG: triacilglicerol; VLDL-c: colesterol em lipoproteínas de muito baixa densidade.

Além disso, observamos alterações na maioria dos biomarcadores dos perfis lipídico e glicêmico nos indivíduos do grupo OE, conforme demonstrado na Figura 2A. Considerando o perfil lipídico tradicional (CT, HDL-c, LDL-c, não HDL-c, VLDL-c, TG, relação CT/HDL-c e relação LDL-c/HDL-c), 44% dos indivíduos no grupo com OE apresentaram um ou mais distúrbios. Os indivíduos do grupo sem OE apresentaram menor frequência (23%; p = 0,004). Quando consideradas as concentrações de Apo A1 e Apo B, mais da metade (54%) dos indivíduos do grupo com OE e 34% do grupo sem OE apresentaram um ou mais distúrbios no perfil lipídico (p = 0,006), conforme mostrado na Figura 2B. A frequência de pelo menos um distúrbio nos biomarcadores do perfil glicêmico foi maior no grupo OE (23%) do que no grupo sem OE (11%) (p = 0,037).

Foi observada diferença entre os grupos com e sem OE em relação ao consumo alimentar de risco apenas para ingestão de gordura total (p = 0,028). No entanto, a prevalência de ingestão inadequada de fibra alimentar e de gordura saturada no grupo com OE foi notável (88,5% para ambos) ( Tabela 2 ).

**Tabela 2:** – Prevalência de consumo de alimentos de risco na amostra total e nos grupos com e sem OE

Variáveis	Amostra total (n=222)		OE (n=157)		Sem OE (n=65)		Valor p
	n	%	n	%	n	%	
VET > necessidade energética	109	49,1	72	45,9	37	56,9	0,134
Proteína > 15% do VET	166	74,8	116	73,9	50	76,9	0,635
Carboidratos > 60% do VET	13	5,8	7	4,5	6	9,2	0,209
Fibra alimentar < 25 g/dia*	192	86,5	139	88,5	53	81,5	0,165
Lipídios > 35% do VET	121	54,5	93	59,2	28	43,1	0,028
AGS ≥ 10% do VET	190	85,6	139	88,5	51	78,5	0,052
AGMI > 15% do VET	42	18,9	33	21,0	9	13,8	0,214
AGPI > 10% do VET	24	10,8	19	12,1	5	7,7	0,336

AGMI: ácidos graxos monoinsaturados; AGPI: ácidos graxos poli-insaturados; AGS: ácidos graxos saturados; OE: obesidade eutrófica; VET: valor energético total.* Ajustado para ingestão de energia. Teste χ ^2^ de Pearson ou teste exato de Fisher.

Para a análise de regressão, foram excluídos 3 indivíduos em razão da falta de dados (n = 219; OE = 154 e sem OE = 65). O modelo final incluiu as seguintes variáveis independentes: sexo, %GC, alterações no perfil lipídico, circunferência do pescoço, IMC e ingestão média de carboidratos ( Tabela 3 ). Para cada 1% de aumento no %GC, houve 3,04 vezes mais chances de pertencer ao grupo com OE, e para cada aumento de 1 cm na circunferência do pescoço, houve 2,52 vezes mais chances de pertencer ao grupo com OE. A OE também foi positivamente associada com a presença de um ou mais distúrbios no perfil lipídico. Houve associações negativas entre OE e sexo feminino e IMC, de modo que para cada aumento de 1 kg/m ^2^ no IMC, observou-se 51% menos chance de pertencer ao grupo com OE.

**Tabela 3:** – Modelo final de regressão logística múltipla ajustado pela estratégia *stepwise* para analisar associações entre a presença da obesidade eutrófica e as variáveis analisadas (n = 219)

	Estimativa (β)	EP	OR (IC 95%)	Valor z	pr (>|t|)	Valor p
Intercepto	-36,183098	9,713135	0,00 (0,00 – 0,00)	-3,725	0,000195	0,001
**Variáveis**						
Sexo feminino	-8,930783	1,948802	0,00 (0,00 – 0,01)	-4,583	<0,0001	0,001
%GC total	1,112949	0,207149	3,04 (2,03 – 4,57)	5,373	<0,0001	**0,001**
Alterações no perfil lipídico (≥ 1)	1,415612	0,699868	4,12 (1,04 – 16,24)	2,023	0,043106	0,050
Circunferência do pescoço	0,924465	0,297740	2,52 (1,41 – 4,52)	3,105	0,001903	0,010
IMC	-0,708130	0,340233	0,49 (0,25 – 0,96)	-2,081	0,037405	0,050
Ingestão média de carboidratos	-0,012656	0,007977	0,99 (0,97 – 1,00)	-1,587	0,112615	1,000

Desvio nulo: 266,362 com 218 graus de liberdade. Desvio residual: 64,027 com 212 graus de liberdade. Número de iterações de pontuação de Fisher: 8. EP: erro padrão; IC: intervalo de confiança; IMC: índice de massa corporal; OR: razão de chances; %GC total: percentual de gordura corporal total. Regressão logística com estimativa de odds ratio e seu respectivo intervalo de confiança de 95%, tendo como desfecho a presença/ausência de obesidade eutrófica.

## Discussão

Este estudo é um dos primeiros realizados no Brasil para avaliar o perfil cardiometabólico de indivíduos jovens com OE em comparação com seus pares sem OE. A prevalência de OE foi significativamente alta. Indivíduos com OE apresentaram piores resultados em relação à composição corporal e biomarcadores cardiometabólicos do que aqueles do grupo sem OE. Além disso, foram encontradas associações significativas entre a presença de OE e fatores de risco cardiometabólicos.

A prevalência muito elevada de OE deve ser interpretada e generalizada com cautela. Além do aumento das taxas de obesidade em todo o mundo, ^38^ o excesso de %GC tem várias consequências prejudiciais, mesmo quando não associado a um aumento do IMC. Em médio e longo prazos, a perda de massa muscular aliada ao alto %GC pode resultar em consequências negativas na qualidade de vida e contribuir para o desenvolvimento da obesidade sarcopênica e outras doenças crônicas não transmissíveis relacionadas à nutrição. ^39 , 40^

Um aspecto importante de nossos resultados é o maior IMC apresentado pelos indivíduos com OE. No entanto, esse resultado poderia ser esperado, uma vez que o IMC apresenta boa correlação com o %GC em diversas populações. Esse resultado demonstra que esses indivíduos, mesmo com maior IMC, poderiam ser classificados como eutróficos, desconsiderando os fatores de risco associados ao excesso de %GC. ^2^ Portanto, é indispensável avaliar e classificar adequadamente os indivíduos com OE.

Outro resultado importante é a baixa faixa etária de nossa amostra, diferente de outros estudos com OE. ^9 , 41 , 42^ A alta prevalência de OE e distúrbios metabólicos em indivíduos jovens traz à tona a importância de se refletir sobre mudanças nos critérios de avaliação e classificação da composição corporal. Os impactos em longo prazo, incluindo as consequências negativas no perfil da composição muscular ao longo do tempo, também precisam ser extensivamente investigados.

A prevalência de %GC aumentada em nosso estudo foi maior do que em um estudo com indivíduos estadunidenses (n = 6.171), que mostrou prevalência de OE de 33,4% tanto para mulheres quanto para homens. ^9^ Também foi maior do que o observado em um estudo com indivíduos chineses (n = 23.748), com uma prevalência de OE de aproximadamente 8%. ^41^ Essa diferença pode ser explicada, em parte, pelos pontos de corte mais altos (33,3% para mulheres e 23,1% para homens e ≥ 24% para homens e ≥ 33% para mulheres nos estudos dos Estados Unidos e da China, respectivamente) e pelo método utilizado para classificação da OE (bioimpedância utilizada em ambos). Madeira et al. ^4^ avaliaram adultos brasileiros com OE e identificaram prevalências menores (9,1%) do que as encontradas em nosso estudo. Os pontos de corte para %GC foram 23,1% para homens e 33,3% para mulheres, e dobras cutâneas foram usadas para a sua medição. Esses resultados reforçam ainda mais a necessidade de estudos para padronizar os pontos de corte e métodos de avaliação da composição corporal, bem como para melhor caracterização da OE. ^43^

Em contraste, um estudo transversal realizado com 1.354 adultos jovens na América Latina encontrou prevalência moderada (29,1%) de OE. Essa condição também foi associada a maior risco cardiovascular, ^44^ corroborando nossos resultados. Esses resultados também enfatizam a importância de cuidados de saúde adequados, considerando que o excesso de %GC pode contribuir progressivamente para um aumento do risco de doenças cardiometabólicas e mortalidade. ^45^

No presente estudo, usamos um valor > 30% ^1^ para classificar as mulheres com presença de OE. No entanto, isso pode ser questionado e exploramos as implicações de aplicar um ponto de corte mais alto, de 32%. ^12^ Verificamos que 14 mulheres não atenderiam aos critérios para classificação da OE, o que resultaria em uma prevalência geral de 64,4%. No entanto, vale ressaltar que a maioria das mulheres que não seriam classificadas com OE apresentou alterações nos perfis lipídico e glicêmico, indicando a presença de fatores de risco associados ao excesso de %GC. Portanto, a utilização de um ponto de corte > 30% garantiria a inclusão de mulheres que apresentam fatores de risco relacionados ao excesso de %GC e poderia auxiliar na identificação indivíduos passíveis de serem beneficiados por intervenções para melhora da saúde metabólica.

Alguns resultados observados nos indivíduos com OE em nosso estudo, tais como IMC mais alto, embora apenas ligeiramente em comparação com a faixa normal, %GC, androide e ginoide %GC e relação androide/ginoide eram esperados, uma vez que o aumento do %GC é a base desta condição. ^1^ No entanto, um maior %GC androide contribui para um risco cardiometabólico aumentado, uma vez que o acúmulo de gordura na região abdominal pode resultar em alterações na função endotelial. ^46^ Além disso, embora nenhum indivíduo tenha apresentado circunferência da cintura fora da faixa normal, os valores mais elevados encontrados em indivíduos com OE indicam uma predisposição a maior risco cardiometabólico. ^19 , 20 , 47^ A circunferência do pescoço também é uma medida importante na avaliação do risco cardiometabólico ^48^ e foi positivamente associada à presença de OE.

Quando avaliamos os biomarcadores tradicionais do perfil lipídico, quase 45% dos indivíduos com OE apresentaram alterações. Em estudo anterior, encontramos uma prevalência de 52,5%. ^10^ Esses altos percentuais de alterações no perfil lipídico, bem como a associação da OE com as concentrações de LDL-c e TG, são importantes para o perfil cardiovascular geral desses indivíduos. O excesso de tecido adiposo visceral suscita o aumento da lipólise de ácidos graxos, que são direcionados ao fígado. No fígado, os ácidos graxos livres são substratos para a síntese de lipoproteínas, incluindo as VLDL. ^48^ Como a VLDL é uma lipoproteína rica em TG, pode haver maior quantidade de ácidos graxos para armazenamento. Além disso, como a captação de ácidos graxos das VLDL pelo tecido adiposo é facilitada pela insulina, ^49^ indivíduos com RI podem desenvolver distúrbios no metabolismo das lipoproteínas. Outras lipoproteínas aterogênicas, como a LDL pequena e densa, também podem se acumular quando o excesso de tecido adiposo é encontrado na região visceral, o que pode piorar o perfil cardiovascular. ^48^

Quando foram avaliadas as concentrações de Apo A1 e B, a frequência de distúrbios no perfil lipídico foi maior em indivíduos com OE do que no grupo sem OE. A Apo A1 é a principal lipoproteína da HDL e desempenha papel importante na remoção do excesso de colesterol dos tecidos. ^50^ A Apo B é amplamente distribuída nas lipoproteínas, ^48^ e durante a RI, sua depuração e a entrada da LDL nas células são comprometidas, o que acelera o processo aterogênico. ^51^ Portanto, a associação negativa entre a presença de OE e as concentrações de Apo A1, bem como a maior frequência de distúrbios nas concentrações de Apo B e no índice HOMA-IR encontrados em indivíduos com OE, são aspectos importantes a serem considerados no estudo desta condição.

A frequência de distúrbios nas concentrações de insulina e no índice HOMA-IR foram semelhantes aos observados em estudo com indivíduos poloneses, no qual, de um total de 168 mulheres e homens, 73 (43%) apresentavam OE. ^12^ O excesso de células adiposas promove disfunção tecidual e ativação de células imunes com potencial atividade pró-inflamatória, desencadeando mediadores pró-inflamatórios locais e sistêmicos, independente do IMC. Consequentemente, há desregulação nas vias de sinalização e ação comprometida da insulina, o que resulta na redução da captação de glicose pelas células e consequente RI. ^48^

Até o momento, apenas alguns estudos avaliaram os hábitos alimentares de indivíduos com OE. ^11 , 44 , 52 , 53^ Männistö et al. ^53^ identificaram fatores dietéticos que podem aumentar o risco de distúrbios cardiometabólicos em indivíduos com OE. Embora não tenha sido avaliado o consumo de gordura saturada, foi encontrada baixa ingestão de fibra alimentar, semelhante aos nossos resultados. O consumo adequado de fibra alimentar promove melhoras no perfil glicêmico, nas concentrações de LDL-c, de não HDL-c e de outras lipoproteínas; diminui o risco de doenças cardiometabólicas; e melhora os perfis inflamatório e imunológico. ^54^ O percentual elevado de consumo excessivo de gorduras totais e saturadas observado em indivíduos com OE deve ser cuidadosamente considerado porque há evidências contundentes sobre a relação entre esses nutrientes e riscos maiores de DCVs. ^55^

Um estudo de base populacional em Teerã (seguimento médio de 18 anos) avaliou a associação entre %GC e risco de mortalidade por DCV em 8.287 indivíduos com mais de 30 anos. Considerando a circunferência da cintura e a relação cintura-quadril como indicadores de adiposidade, indivíduos com peso normal e com obesidade central apresentaram risco aumentado de mortalidade por todas as causas e por DCV em comparação com indivíduos com peso normal e sem obesidade central. Embora os autores tenham usado apenas indicadores indiretos para avaliar o %GC, os resultados reforçam a importância de avaliar os riscos cardiometabólicos em adultos com excesso de %GC. ^56^

Entre os pontos fortes do nosso estudo, é importante mencionar que usamos DXA para avaliar a composição corporal, que é o método padrão-ouro. Além disso, a análise das concentrações de apolipoproteínas forneceu dados mais robustos em relação ao perfil cardiometabólico. Também é pertinente mencionar a homogeneidade nos dados socioeconômicos e de estilo de vida dos grupos avaliados, o que reduz possíveis vieses. Além disso, garantimos que fossem excluídos do estudo indivíduos com condições médicas preexistentes ou práticas de estilo de vida não convencionais que poderiam introduzir interpretações e generalizações tendenciosas. Por outro lado, uma limitação importante para a discussão de nossos resultados depende dos diferentes pontos de corte e métodos de avaliação do %GC encontrados em outros estudos, bem como da falta de pontos de corte padronizados para a população brasileira. Vale ressaltar que o estudo identificou fatores de risco associados a adultos jovens assintomáticos e saudáveis. Isso destaca a importância de fornecer atenção e cuidados de saúde adequados a esses indivíduos na prática clínica, visto que podem apresentar risco aumentado de desfechos adversos à saúde, inicialmente sem sintomas.

## Conclusão

Os indivíduos com OE apresentaram pior perfil cardiometabólico do que aqueles sem OE. A OE foi associada a importantes variáveis antropométricas, de composição corporal e cardiometabólicas. De modo geral, esses resultados corroboram aqueles relatados em alguns estudos prévios. No entanto, estudos futuros sobre a padronização de pontos de corte e métodos de avaliação da composição corporal em diferentes populações são de extrema importância. A avaliação e interpretação precisas da composição corporal, independentemente do IMC, são cruciais na prática clínica para facilitar o manejo eficaz das comorbidades de médio e longo prazos associadas ao excesso de %GC. Essas avaliações auxiliarão os profissionais de saúde a reduzir os riscos de complicações cardiometabólicas negativas, fornecendo estratégias de gerenciamento personalizadas para indivíduos com OE.
