# Supplementary material for: Associações entre Obesidade Eutrófica e Alterações no Perfil Lipídico de Adultos Jovens
Source: Arq Bras Cardiol. 2023 Oct 6;120(9):e20220914. [Article in Portuguese] doi: 10.36660/abc.20220914 (PMC10567082; doi:10.36660/abc.20220914)
Supplement: Translation

# Translation: Associations between Normal-Weight Obesity and Disturbances in the Lipid Profile of Young Adults

**Authors:** Anna Flavia Ferreira Passos, Acsa de Castro Santos, Alexandre Siqueira Guedes Coelho, Cristiane Cominetti

**Affiliations:** 1 Brazil Grupo de Pesquisa em Genômica Nutricional, Faculdade de Nutrição, Universidade Federal de Goiás, Goiânia, GO – Brazil; 2 Brazil Faculdade de Agronomia, Universidade Federal de Goiás, Goiânia, GO – Brazil

**Keywords:** Apolipoproteins, Body Composition, Dyslipidemias, Cardiovascular Diseases, Blood Glucose

## Abstract

**Background:**

Normal-weight obesity (NWO) is characterized by normal body mass index (BMI) but high body fat percentage (%BF) that increases the risks of cardiometabolic comorbidities. Accurate assessment and interpretation of body composition data are necessary to reduce these risks.

**Objectives:**

To compare the cardiometabolic profile of individuals with NWO and normal %BF and evaluate the associated risk factors.

**Methods:**

A cross-sectional study was conducted with 222 Brazilian adults from a university community, of whom 157 had NWO and 65 had normal BMI and %BF (non-NWO). All participants reported being asymptomatic and without underlying health conditions. Socioeconomic, lifestyle, food intake, anthropometry, body composition measures (using dual-energy radiological absorptiometry), and lipid and glycemic profiles were evaluated. A p < 0.05 was established as significant.

**Results:**

The median age of the participants was 23 years (interquartile range: 21 to 25), and most were female (67.1%). No significant differences were found in blood pressure, age, or physical activity levels between the NWO and non-NWO groups. However, the frequency of lipid profile disturbances was higher in the NWO group (54%) compared to the non-NWO group (34%) (p < 0.006). Neck circumference, %BF, and lipid profile disturbances were positively associated with NWO.

**Conclusion:**

Individuals with NWO have a worse cardiometabolic profile than those without NWO, and this condition is associated with important biomarkers. Addressing these outcomes is important for preventing long-term cardiometabolic complications. Accurate assessment and interpretation of body composition data, regardless of BMI, are crucial in this scenario.

## Introduction

Normal-weight obesity (NWO), which was described in 2006, is a condition characterized by normal body mass index (BMI) and high body fat percentage (%BF). ^1^ It was described because of failures in the concepts of BMI and obesity. Obesity is defined by the World Health Organization as an excess of body fat associated with health risks ^1 , 2^ and is diagnosed based on BMI. However, BMI is known to be a flawed index, especially when applied at the individual level, as it does not differentiate between lean body mass and fat body mass. ^2 , 3^

As a result of excess %BF, individuals with NWO are at a higher risk of developing metabolic syndrome, atherogenic dyslipidemia, obesity, and cardiometabolic diseases, including type 2 diabetes mellitus and cardiovascular diseases (CVDs), compared to individuals with normal %BF. ^1 , 3 - 5^ CVDs are the leading cause of death worldwide and are projected to account for over 23 million deaths by 2030. ^6^

Therefore, considering the importance of adiposity as a risk factor for CVDs and other nutrition-related chronic non-communicable diseases, the assessment of %BF in individuals with normal BMI is crucial for accurate diagnosis and early interventions. ^7 , 8^ It is estimated that approximately 30 million people from the United States have NWO, ^9^ and a few Brazilian studies on this subject ^4 , 10 , 11^ have revealed that NWO is associated with metabolic syndrome and insulin resistance (IR). ^4^ In our previous study, we showed a high frequency of dyslipidemia in individuals with NWO. ^10^ These results reinforce the relationship between NWO and disturbances in lipid and glycemic biomarkers, which are important predictors of CVDs and type 2 diabetes mellitus development.

Driven by the hypothesis that high %BF can lead to a disturbed cardiometabolic profile even in the presence of normal BMI and the importance of understanding the metabolic aspects of NWO, this study aimed to compare the cardiometabolic profile of individuals with and without NWO, as well as to evaluate the associations with socioeconomic, anthropometric, body composition, biochemical, and food consumption data.

## Methods

### Study design and participants

This was a cross-sectional study with recruitment and data collection from January to June 2019. The study was disseminated through folders, social networks, and e-mails sent to students, professors, and other employees at the Federal University of Goiás. Individuals who presented with a normal BMI (between 18.50 and 24.99 kg/m ^2^ ) ^2^ and aged between 20 and 59 years were included. Smokers; individuals with metal implants or limb amputation; individuals with intense physical activity (athletes or regular practitioners of high-performance exercise); self-reported vitamin and/or mineral supplementation; self-reported acute and/or chronic diseases; self-reported use of lipid-lowering drugs, anti-hypertensives, anti-glycemic drugs, or insulin; pregnant or lactating women; women in menopause or undergoing hormone replacement therapy; individuals undergoing nutritional monitoring and/or who changed their usual diet in the last 6 months prior to data collection; and those who missed some stage of data collection were not included, as described in Figure 1 . These exclusion criteria were implemented to manage potential confounding variables that may introduce bias in the relationship between excess body fat and the variables under investigation in this study.

**Figure 1:** – Flow-chart of participation recruitment

Individuals were grouped according to sex and age, and the cutoff points used in two well-established studies on NWO were applied to classify %BF. The criteria for cutoff points were selected based on consistency in the method of body composition assessment. Initially, the reference by the authors who originally defined NWO ^1^ was utilized for women; however, they did not provide cutoff points for men. To address this gap, a male reference with a more sensitive cutoff point that still captured associated risk factors was sought. ^12^ For women and men, cutoff points > 30% ^1^ and > 19%, ^12^ respectively, were applied to classify high %BF. Given the lack of a standardized criterion for classifying the prevalence of NWO, we adopted a set of proposed thresholds for overweight and obesity in adults, considering the association between excess %BF and health risks. ^1 , 2^ The proposed limits for classification were determined as follows: < 20% for very low, 20% to < 30% for low, 30% to < 50% for moderate, 50% to < 70% for high, and ≥ 70% for very high. ^13^

### Data collection

Data were collected at the School of Nutrition and the Clinical Research Unit of the Federal University of Goiás Teaching Hospital. First, the participants received information about the study and signed an informed consent form. A questionnaire was administered to assess socioeconomic, demographic, health, lifestyle, and food consumption data (one 24-hour dietary recall). Three non-consecutive measurements of blood pressure, ^14^ dual-energy radiological absorptiometry (DXA), and anthropometric evaluation ^15^ were conducted. Economic classification ^16^ and physical activity level ^17^ were also determined.

Blood samples were collected from the median cubital vein after a 12-hour fast by a qualified professional. Immediately after collection, blood samples were transferred to appropriate tubes to obtain serum and/or plasma and then sent to the laboratory for biochemical analyses. Individuals were instructed not to consume alcoholic beverages or engage in intense physical activity within 72 hours prior to blood collection. They were instructed to maintain their usual diet and maintain a stable weight in the last two weeks prior to blood collection. ^18 - 20^ Two other non-consecutive 24-hour dietary recalls, including one weekend day, were collected. ^21^

### Anthropometry and body composition

Body mass was measured on a Filizola ^®^ digital scale (Filizola Shop, São Paulo, Brazil), and height was determined using a Seca ^®^ stadiometer (Seca Deutschland, Hamburg, Germany). ^15^ Waist and neck circumferences were measured in duplicate with a 200 cm long and 1 mm accurate Seca ^®^ body measure tape (Seca Deutschland, Hamburg, Germany), and the mean value was used for data analysis. Body composition was measured using a DPX NT Lunar ^®^ DXA device (General Electric Medical Systems, Madison, USA). ^22^

### Cardiometabolic biomarkers

Blood concentrations of total cholesterol (TC), triacylglycerol (TG), low-density lipoprotein cholesterol (LDL-C), high-density lipoprotein cholesterol (HDL-C), very low-density lipoprotein cholesterol (VLDL-C), apolipoprotein (Apo) A1, Apo B, glucose, insulin, and glycated hemoglobin (HbA _1c_ ) were evaluated. Serum lipid profile biomarkers were determined using a direct colorimetric enzymatic method. The LDL-C, VLDL-C, and non-HDL-C concentrations were estimated using equations. ^19 , 21^ The following cutoffs were used to classify the markers as altered: TC ≥ 190, LDL-C ≥ 130, non-HDL-C ≥ 160, TG ≥ 150, VLDL-C ≥ 30, HDL-C < 40 (all in mg/dL); TC:HDL-C ratio ≥ 4.4 for women and ≥ 5.1 for men; and LDL-C:HDL-C ratio ≥ 2.9 for women and ≥ 3.3 for men. ^19 , 23 - 25^ Apo A1 and Apo B concentrations were analyzed using the turbidimetry method and were considered altered when Apo A1 was < 140 mg/dL for women and < 120 mg/dL for men ^22^ and when Apo B was ≥ 104 mg/dL for women and men. ^26^ The Apo B:Apo A1 ratio was considered elevated when it was ≥ 0.6 for women and ≥ 0.7 for men. ^27 , 28^ The atherogenic index was estimated, and values were considered high when > 2.24. ^29^

Serum glucose concentrations were determined using the enzymatic colorimetric method, and the reference values established by the Brazilian Society of Diabetes were adopted. ^20^ Total blood HbA _1c_ concentrations were evaluated with the immunoturbidimetric inhibition method, and the Brazilian Society of Diabetes reference values were applied. ^20^ Serum insulin concentrations were assessed using electrochemiluminescence. Homeostasis model assessment of IR (HOMA-IR), HOMA2-IR, HOMA of beta-cell function (HOMA-beta), and quantitative insulin sensitivity check (QUICKI) indices were calculated. ^20 , 30^ The cutoff points were > 2.71 for HOMA-IR, > 1.80 for HOMA2-IR, above the ninetieth percentile of the sample for HOMA-beta, and lower than the tenth percentile of the sample for the QUICKI index. ^20 , 30^ The triacylglycerol-glucose (TyG) index was estimated, and values higher than 4.55 for women and 4.68 for men were used as cutoff points. ^20^

### Food consumption

Three 24-hour dietary recalls were applied on non-consecutive days, including one weekend day, ^20^ following the multiple pass method. ^31^ Two trained nutritionists applied one face-to-face 24-hour dietary recall and two 24-hour dietary recall via phone calls. A photographic manual and household measuring utensils were used to aid in the quantification of food portions. ^32^ Conversion of household measures into grams ^33^ and data management were standardized. The 24-hour dietary recalls were evaluated using Nutrition Data System for Research software (NDSR, Nutrition Coordinating Center, University of Minnesota, Minneapolis–Saint Paul, USA). ^34^ The values proposed by the Brazilian Society of Cardiology were applied for macronutrients, dietary fiber, and fatty acid adequacy. ^19^

### Statistical analysis and justification of sample size

Double-entry databases were built to check for consistency. Despite the selection criteria for the adult age group from 20 to 59 years old according to the World Health Organization, with the recruitment of only two adults over 40 years old, these were excluded from the analyses, remaining only young adults aged 39 years old or less, so that in terms of age, the sample did not show these outliers. Considering that there are no studies with representative samples of Brazil, the sample calculation was based on Cohen’s effect size for independent samples. ^35^ The statistical power to reject the null hypothesis was set at 80%, with a type I error probability of 0.05, and sample sizes of 157 and 65 observations for NWO and non-NWO, respectively. The estimated effect size was 0.41, which is considered medium. ^35^

Data distribution was assessed using the Shapiro-Wilk test. Either an unpaired Student’s t test or a Mann-Whitney test was used for comparison, based on the data distribution. Analysis of categorical data and frequency of disturbances in the cardiometabolic profile of individuals with and without NWO was performed using either the χ ^2^ test or Fisher’s exact test. Nutrient intake data were energy-adjusted when necessary. ^36^ Categorical variables were presented as absolute and relative frequencies (%), and continuous variables were presented as mean ± standard deviation or median (interquartile range) according to data distribution.

We employed multiple logistic regression models to investigate the relationships between independent and dependent variables. To enhance the models, we used the stepwise strategy, an automated procedure for selecting the most significant predictor variables to include in the model. Independent variables were sex; skin color; age; physical activity level; systolic and diastolic blood pressures; weight; height; BMI; neck and waist circumferences; %BF; gynoid and android %BF; android to gynoid %BF ratio; fasting blood glucose and insulin; HOMA-IR; HOMA-2IR; HOMA-beta; HbA _1c_ ; QUICKI index; Apo A1; Apo B; TC; HDL-C; LDL-C; non-HDL-C; VLDL-C; TG; TC:HDL-C, LDL-C:HDL-C, and Apo B:Apo A1 ratios; TyG; atherogenic index; presence of disturbances in glycemic and lipid profiles; energy; total fat; carbohydrate; protein; dietary cholesterol; saturated, mono-, and polyunsaturated fatty acids; and dietary fiber intake. All variables were chosen according to their clinical importance with the studied condition. P < 0.05 was considered statistically significant, and all analyses were performed using R software version 4.0.3. ^37^

## Results

A total of 222 individuals were recruited ( Figure 1 ), 67% of whom were women. The study participants were aged between 20 and 34 years and had normal BMI. Following the classification of %BF, 157 (71%) participants were assigned to the NWO group, indicating a high prevalence, while the remaining 65 participants were assigned to the non-NWO group. When considering sex, 72.5% of women and 67.1% of men showed increased %BF. Regarding the cutoff point for %BF classification, we used a value > 30% ^1^ to classify women as having NWO. However, because this cutoff point can be questioned, we examined the implications of using a cutoff point greater than 32% ^12^ and found that 14 women would not meet the criteria for the NWO classification. As a result, the total number of individuals classified as having NWO would be reduced to 143, resulting in an overall prevalence of 64.4%, still considered high. However, out of the 14 women who would not be classified as having NWO, 11 exhibited disturbances in lipid and glycemic profiles, indicating the presence of risk factors associated with excess %BF.

Table 1 describes socioeconomic, lifestyle, anthropometric, body composition, and biochemical data of individuals in the NWO and the non-NWO groups. Individuals in the NWO group showed higher values of weight, BMI, waist circumference, %BF, android and gynoid %BF, android to gynoid ratio, insulin, HOMA-IR, HOMA-beta, TyG, TC, LDL-C, non-HDL-C, VLDL-C, TG, TC:HDL-C ratio, LDL-C:HDL-C ratio, Apo B, and Apo B:Apo A1 ratio, as well as lower QUICKI index values than those in the non-NWO group.

**Table 1:** – Socioeconomic, lifestyle, anthropometric, body composition, and biochemical variables of the total sample and the NWO and non-NWO groups

Variables	Total (n = 222, 100.0%)	NWO (n = 157, 70.7%)	Non-NWO (n = 65, 29.3%)	p value
**Sex**				0.410
Male	73 (32.9)	49 (31.2)	24 (36.9)	
Female	149 (67.1)	108 (68.8)	41 (63.1)	
**Skin color**				0.522
White	84 (37.8)	64 (40.8)	20 (30.8)	
Brown	94 (42.3)	63 (40.1)	31 (47.7)	
Black	29 (13.1)	19 (12.1)	10 (15.4)	
Asian	15 (6.8)	11 (7.0)	4 (6.2)	
**Marital status**				0.522
Single	199 (89.6)	138 (87.9)	61 (93.8)	
Married	22 (9.9)	18 (11.5)	4 (6.2)	
Divorced	1 (0.5)	1 (0.6)	0 (0)	
**Education level**				0.065
Higher incomplete	167 (75.2)	114 (72.6)	53 (81.5)	
Higher complete	44 (19.8)	32 (20.4)	12 (18.5)	
Graduation complete	11 (5.0)	11 (7.0)	0 (0)	
**Socioeconomic class**				0.094
Higher	143 (64.4)	106 (67.5)	37 (56.9)	
Intermediate	78 (35.1)	51 (32.5)	27 (41.5)	
Lower	1 (0.5)	0 (0)	1 (1.5)	
Age (years)	23.0 (21.0 – 25.0)	23.0 (21.0 – 26.0)	23.0 (21.0 – 24.0)	0.506
SBP (mmHg)	109.5 (100.0 – 115.8)	109.5 (100.0 – 116.0)	110.0 (100.0 – 115.0)	0.682
DBP (mmHg)	67.0 (60.1 – 74.0)	66.5 (60.0 – 74.0)	70.0 (62.0 – 74.0)	0.349
Weight (kg)	60.6 (55.0 – 66.6)	61.3 (56.0 – 66.7)	57.4 (53.0– 65.9)	0.026
Height (m)	1.7 (1.6 – 1.7)	1.7 (1.6 – 1.7)	1.7 (1.6 – 1.7)	0.719
BMI (kg/m ^2^ )	21.8 (20.5 – 23.0)	22.1 (21.0 – 23.3)	20.8 (19.8 – 22.0)	<0.0001
PA level (MET min/week)	260.0 (131.2 – 409.8)	240.0 (100.0 – 415.0)	280 (180.0 – 409.0)	0.210
NC (cm)	32.4 (31.2 – 36.0)	32.5 (31.4 – 35.8)	32.1 (30.7 – 36.1)	0.490
WC (cm)	72.7 ± 5.5	73.8 ± 5.2	70.1 ± 5.4	<0.0001
%BF DXA	30.4 (23.4 – 36.4)	34.2 (28.8 – 37.7)	24.6 (15.4 – 28.1)	<0.0001
Android %BF	30.7 ± 9.6	34.7 ± 7.6	20.9 ± 6.4	<0.0001
Gynoid %BF	42.8 (34.2 – 48.4)	46.5 (38.1 – 49.5)	36.8 (23.2 – 41.0)	<0.0001
A:G	0.8 (0.7 – 0.9)	0.8 (0.7 – 0.9)	0.6 (0.6 – 0.8)	<0.0001
Glucose (mg/dL)	85.0 (80.2 – 89.8)	85.0 (81.0 – 90.0)	84.0 (80.0 – 89.0)	0.392
Insulin (pmol/L)	49.9 (38.9 – 66.6)	54.5 (42.4 – 71.8)	43.0 (35.2 – 54.5)	0.0001
HOMA-IR	1.4 (1.1 – 2.0)	1.6 (1.2 – 2.1)	1.2 (1.0 – 1.6)	0.0001
HOMA2-IR	0.9 (0.7 – 1.2)	1.0 (0.8 – 1.3)	0.8 (0.6 – 1.0)	0.0001
HOMA-beta	122.0 (87.9 – 170.1)	134.0 (95.1 – 176.1)	104.5 (78.9 – 149.6)	0.011
HbA _1c_ %	4.8 (4.6 – 5.0)	4.8 (4.6 – 5.0)	4.8 (4.6 – 5.0)	0.953
QUICKI Index	0.36 ± 0.02	0.36 ± 0.02	0.37 ± 0.02	<0.0001
TC (mg/dL)	175.7 ± 32.0	179.8 ± 33.1	165.8 ± 26.9	0.002
HDL-C (mg/dL)	55.0 (47.0 – 65.0)	55.0 (47.0 – 65.0)	56.0 (48.0 – 65.0)	0.782
LDL-C (mg/dL)	99.0 (82.0 – 117.8)	103.0 (83.0 – 118.0)	93.0 (74.0 – 107.0)	0.008
Non-HDL-C (mg/dL)	115.0 (96.2 – 136.0)	121.0 (100.0 – 140.0)	109.0 (90.0 – 121.0)	0.002
VLDL-C (mg/dL)	14.8 (10.6 – 20.8)	16.4 (11.4 – 21.8)	12.8 (9.6 – 16.6)	0.001
TG (mg/dL)	74.0 (53.2 – 104.0)	82.0 (57.0 – 109.0)	64.0 (48.0 – 83.0)	0.001
TC:HDL-C ratio	3.0 (2.6 – 3.6)	3.1 (2.7 – 3.8)	2.8 (2.5 – 3.3)	0.021
LDL-C:HDL-C ratio	1.8 (1.4 – 2.3)	1.8 (1.4 – 2.4)	1.7 (1.3 – 2.0)	0.050
Apo A1 (mg/dL)	151.0 (137.0 – 168.0)	151.5 (138.8 – 172.2)	149.0 (137.0 – 163.0)	0.246
Apo B (mg/dL)	76.0 (65.0 – 89.0)	80.4 ± 19.0	70.6 ± 14.8	0.0003
Apo B:Apo A1 ratio	0.5 (0.4 – 0.6)	0.5 (0.4 – 0.6)	0.5 (0.4 – 0.5)	0.031
TyG	3.5 ± 0.2	3.5 ± 0.2	3.4 ± 0.2	0.001
AI	1.0 (0.7 – 1.5)	1.0 (0.8 – 1.7)	0.9 (0.6 – 1.2)	0.022

Data are presented as mean ± standard deviation, median (interquartile range), or absolute and relative frequencies. A:G: ratio between android and gynoid %BF; AI: atherogenic index; Apo A1: apolipoprotein A1; Apo B: apolipoprotein B; android %BF: percentage of android body fat; BMI: body mass index; DBP: diastolic blood pressure; gynoid %BF: percentage of gynoid body fat; HbA _1c_: glycated hemoglobin; HDL-C: high-density lipoprotein cholesterol; HOMA-beta: homeostatic model assessment of beta cell function; HOMA-IR: homeostasis model assessment of insulin resistance; HOMA2-IR: homeostasis model assessment 2 of insulin resistance; LDL-C: low-density lipoprotein cholesterol; MET: metabolic equivalents; NC: neck circumference; non-HDL-C: non-HDL cholesterol; NWO: normal-weight obesity; PA: physical activity level; %BF DXA: percentage of body fat assessed by dual-energy radiological absorptiometry; SBP: systolic blood pressure; TC: total cholesterol; TG: triacylglycerol; TyG: triacylglycerol glucose index; VLDL-C: very low-density lipoprotein cholesterol; WC: waist circumference. Significant differences between groups: Student’s t test or Mann-Whitney test, Pearson’s χ ^2^ test or Fisher’s exact test.

Furthermore, we observed disturbances in most biomarkers of lipid and glycemic profiles in individuals in the NWO group, as shown in Figure 2A. Considering the traditional lipid profile (TC, HDL-C, LDL-C, non-HDL-C, VLDL-C, TG; TC:HDL-C and LDL-C:HDL-C ratios), 44% of the individuals in the NWO group showed one or more disturbances. Individuals in the non-NWO group showed a lower frequency (23%; p = 0.004). When Apo A1 and Apo B concentrations were considered, more than half (54%) of the individuals in the NWO group and 34% of those in the non-NWO group showed one or more disturbances in the lipid profile (p = 0.006), as shown in Figure 2B. The frequency of at least one disturbance in the biomarkers of the glycemic profile was higher in the NWO group (23%) than in the non-NWO group (11%) (p = 0.037).

**Figure 2:** – A) Frequency of lipid and glycemic profile disturbances in the NWO and non-NWO groups. B) Frequency of at least one lipid or glycemic profile disturbance in the NWO and non-NWO groups. AI: atherogenic index; Apo A1: apolipoprotein A1; Apo B: apolipoprotein B; HbA1c: glycated hemoglobin; HDL-C: high-density lipoprotein cholesterol; HOMA-beta: homeostatic model assessment of beta cell function; HOMA-IR: homeostasis model assessment of insulin resistance; HOMA2-IR: homeostasis model assessment 2 of insulin resistance; LDL-C: low-density lipoprotein cholesterol; non-HDL-C: non-HDL cholesterol; NWO: normal-weight obesity; TC: total cholesterol; TG: triacylglycerol; VLDL-C: very low-density lipoprotein cholesterol.

A difference between the NWO and non-NWO groups regarding risky food consumption was observed only for total fat intake (p = 0.028). However, the prevalence of inadequate intake of dietary fiber and saturated fat in the NWO group was noteworthy (88.5% for both) ( Table 2 ).

**Table 2:** – Prevalence of risky food consumption in the total sample and the NWO and non-NWO groups

Variables	Total sample (n=222)		NWO (n=157)		Non-NWO (n=65)		p value
	n	%	n	%	n	%	
TEI > energy requirement	109	49.1	72	45.9	37	56.9	0.134
Protein > 15% of TEI	166	74.8	116	73.9	50	76.9	0.635
Carbohydrates > 60% of TEI	13	5.8	7	4.5	6	9.2	0.209
Dietary fiber < 25 g/day*	192	86.5	139	88.5	53	81.5	0.165
Lipids > 35% of TEI	121	54.5	93	59.2	28	43.1	0.028
SFA ≥ 10% of TEI	190	85.6	139	88.5	51	78.5	0.052
MUFA > 15% of TEI	42	18.9	33	21.0	9	13.8	0.214
PUFA > 10% of TEI	24	10.8	19	12.1	5	7.7	0.336

MUFA: monounsaturated fatty acids; NWO: normal-weight obesity; PUFA: polyunsaturated fatty acids; SFA: saturated fatty acids; TEI: total energy intake. *Adjusted for energy intake. Pearson’s χ ^2^ test or Fisher’s exact test.

For the regression analysis, 3 individuals were excluded due to missing data (n = 219; NWO = 154 and non-NWO = 65). The final model included the following independent variables: sex, %BF, disturbances in lipid profile, neck circumference, BMI, and mean carbohydrate intake ( Table 3 ). For each 1% increase in %BF, there were 3.04 times higher odds of belonging to the NWO group, and for each 1 cm increase in neck circumference, there were 2.52 times higher odds of belonging to the NWO group. NWO was also positively associated with the presence of one or more disturbances in the lipid profile. There were negative associations between NWO and female sex and BMI, such that for each 1 kg/m ^2^ increase in BMI, there was 51% lower odds of belonging to the NWO group.

**Table 3:** – Final multiple logistic regression model adjusted by the stepwise strategy to analyze associations between the presence of normal-weight obesity and the analyzed variables (n = 219)

	Estimate (β)	SE	OR (95% CI)	z value	pr (>|t|)	p value
Intercept	-36.183098	9.713135	0.00 (0.00 – 0.00)	-3.725	0.000195	0.001
**Variables**						
Female sex	-8.930783	1.948802	0.00 (0.00 – 0.01)	-4.583	<0.0001	0.001
Total %BF	1.112949	0.207149	3.04 (2.03 – 4.57)	5.373	<0.0001	**0.001**
Disturbances in lipid profile (≥1)	1.415612	0.699868	4.12 (1.04 – 16.24)	2.023	0.043106	0.050
Neck circumference	0.924465	0.297740	2.52 (1.41 – 4.52)	3.105	0.001903	0.010
BMI	-0.708130	0.340233	0.49 (0.25 – 0.96)	-2.081	0.037405	0.050
Mean carbohydrate intake	-0.012656	0.007977	0.99 (0.97 – 1.00)	-1.587	0.112615	1.000

Null deviance: 266.362 with 218 degrees of freedom. Residual deviance: 64.027 with 212 degrees of freedom. Number of Fisher scoring iterations: 8. BMI: body mass index; CI: confidence interval; OR: odds ratio; %BF total: percentage of total body fat; SE: standard error. Logistic regression with an estimate of odds ratio and its respective 95% CI, using the presence/absence of normal-weight obesity as the outcome.

## Discussion

This study is one of the first performed in Brazil to assess the cardiometabolic profile of young individuals with NWO compared with their non-NWO counterparts. The prevalence of NWO was significantly high. Individuals with NWO showed worse results regarding body composition and cardiometabolic biomarkers than those in the non-NWO group. Furthermore, significant associations between the presence of NWO and cardiometabolic risk factors were found.

The very high prevalence of NWO should be cautiously interpreted and generalized. In addition to increasing obesity rates worldwide, ^38^ excess %BF has several harmful consequences, even when not associated with an increased BMI. In the medium- and long-terms, loss of muscle mass combined with high %BF can lead to negative consequences on quality of life and contribute to the development of sarcopenic obesity and other nutrition-related chronic non-communicable diseases. ^39 , 40^

An important aspect of our results is the higher BMI presented by individuals with NWO. However, this result could be expected, since BMI presents a good correlation with %BF in several populations. This result demonstrates that these individuals, even with a higher BMI, could be classified as normal-weight, disregarding the risk factors associated with excess %BF. ^2^ Therefore, it is imperative to evaluate and properly classify individuals with NWO.

Another important finding is the low age range of our sample, which is different from that of other studies with NWO. ^9 , 41 , 42^ The high prevalence of NWO and metabolic disturbances in young individuals raises the importance of reflecting on changes in assessment criteria and classification of body composition. The long-term impacts, including the negative consequences on the muscle composition profile over time, also need to be extensively investigated.

The prevalence of increased %BF in our study was higher than that in a study of individuals from the United States (n = 6,171), which showed an NWO prevalence of 33.4% for both women and men. ^9^ It was also higher than that observed in a study of Chinese individuals (n = 23,748), with an NWO prevalence of approximately 8%. ^41^ This difference can be partly explained by the higher cutoff points (33.3% for women and 23.1% for men and ≥ 24% for men and ≥ 33% for women in the studies from the United States and China, respectively) and by the method used for the classification of NWO (bioimpedance used in both). Madeira et al. ^4^ evaluated Brazilian adults with NWO and identified lower prevalence rates (9.1%) than those found in our study. The cutoff points for %BF were 23.1% for men and 33.3% for women, and skinfolds were used to measure it. These results further underscore the need for studies to standardize the cutoff points and methods for assessing body composition, as well as to better characterize NWO. ^43^

In contrast, a cross-sectional study conducted with 1,354 young adults in Latin America found a moderate prevalence (29.1%) of NWO. This condition was also associated with a higher cardiovascular risk, ^44^ corroborating our results. These results also emphasize the importance of proper healthcare, considering that excess %BF can progressively contribute to an increased risk of cardiometabolic diseases and mortality. ^45^

In this study, we used a value > 30% ^1^ to classify women as having NWO. However, this can be questioned, and we explored the implications of using a higher cutoff point of 32%. ^12^ We found that 14 women would not meet the criteria for NWO classification, resulting in an overall prevalence of 64.4%. Nevertheless, it is noteworthy that the majority of the women who would not be classified as having NWO exhibited disturbances in lipid and glycemic profiles, indicating the presence of risk factors associated with excess %BF. Therefore, the use of a cutoff point > 30% would ensure the inclusion of women who have risk factors related to excess %BF and may help to identify individuals who could benefit from interventions to improve their metabolic health.

Some results observed in individuals with NWO in our study, such as higher BMI, although only slightly compared to the normal range, %BF, android and gynoid %BF, and android to gynoid ratio were expected, given that increased %BF is the foundation of this condition. ^1^ Nevertheless, higher android %BF contributes to an increased cardiometabolic risk since the accumulation of fat in the abdominal region can result in alterations in endothelial function. ^46^ In addition, although no individual had waist circumference outside the normal range, the higher values found in individuals with NWO indicate a predisposition to higher cardiometabolic risk. ^19 , 20 , 47^ Neck circumference is also an important measure in the assessment of cardiometabolic risk ^48^ and was positively associated with the presence of NWO.

When we evaluated traditional biomarkers, almost 45% of individuals with NWO showed disturbances in their lipid profiles. In a previous study, we found a prevalence of 52.5%. ^10^ These high percentages of disturbances in the lipid profile, as well as the association of NWO with LDL-C and TG concentrations, are important to the overall cardiovascular profile of these individuals. Excess visceral adipose tissue leads to increased lipolysis of fatty acids, which are supplied to the liver. In the liver, free fatty acids are substrates for the synthesis of lipoproteins, including VLDL. ^48^ As VLDL is a TG-rich lipoprotein, there may be a higher amount of fatty acids for storage. In addition, because the uptake of fatty acids from VLDL by adipose tissue is facilitated by insulin, ^49^ individuals with IR may develop disturbances in lipoprotein metabolism. Other atherogenic lipoproteins, such as small and dense LDL, can also accumulate when excess adipose tissue is found in the visceral region, thereby worsening the cardiovascular profile. ^48^

When Apo A1 and B concentrations were evaluated, the frequency of disturbances in the lipid profile was higher in individuals with NWO than in the non-NWO group. Apo A1 is the main lipoprotein of HDL, and it plays an important role in removing excess cholesterol from tissues. ^50^ Apo B is widely distributed in lipoproteins, ^48^ and during IR, its clearance and LDL entry into cells are impaired, which accelerates the atherogenic process. ^51^ Therefore, the negative association between NWO and Apo A1 concentrations, as well as the higher frequency of disturbances in Apo B concentrations and the HOMA-IR index found in individuals with NWO, are important aspects to be considered in the study of this condition.

The frequency of disturbances in insulin concentrations and HOMA-IR were similar to those observed in a study of Polish individuals, in which, from a total of 168 women and men, 73 (43%) had NWO. ^12^ Excess adipose cells promote tissue dysfunction and activation of immune cells with potential proinflammatory activity, triggering local and systemic proinflammatory mediators, regardless of BMI. Consequently, there is a deregulation in signaling pathways and an impaired insulin action, which results in reduced glucose uptake by cells and subsequent IR. ^48^

To date, only a few studies have evaluated the dietary habits of individuals with NWO. ^11 , 44 , 52 , 53^ Männistö et al. ^53^ identified dietary factors that may increase the risk of cardiometabolic disturbances in individuals with NWO. Although the consumption of saturated fat was not evaluated, a low intake of dietary fiber was found, which is similar to our results. Adequate consumption of dietary fiber promotes improvements in glycemic profile, LDL-C, non-HDL-C, and other lipoprotein concentrations; lowers the risk of cardiometabolic diseases; and improves inflammatory and immune profiles. ^54^ The high percentage of excessive consumption of total and saturated fats observed in individuals with NWO must be carefully managed because there is overwhelming evidence on the relationship between these nutrients and higher risks of CVDs. ^55^

A population-based study in Tehran (median follow-up of 18 years) evaluated the association between %BF and the risk of mortality from CVD in 8,287 individuals older than 30 years. Considering waist circumference and waist-to-hip ratio as adiposity indicators, normal-weight individuals with central obesity showed an increased risk of all-cause and CVD mortality compared with normal-weight and non-centrally obese individuals. Although the authors used only indirect indicators to assess %BF, the results underscore the importance of assessing cardiometabolic risks in adults with excess %BF. ^56^

Among the strengths of our study, it is important to mention that we used DXA to evaluate body composition, which is the gold-standard method. In addition, the analysis of apolipoprotein concentrations provided more robust data regarding the cardiometabolic profile. It is also pertinent to mention the homogeneity in the socioeconomic and lifestyle data of the groups evaluated, which reduces possible bias. In addition, we took great care to ensure that individuals with preexisting medical conditions or unconventional lifestyle practices that could potentially introduce biased interpretations and generalizations were excluded from the study. On the other hand, an important limitation to the discussion of our results relies on the different cutoff points and methods for the evaluation of %BF found in other studies, as well as the lack of standardized cutoff points for the Brazilian population. It is noteworthy that the study identified risk factors associated with asymptomatic, healthy young adults. This highlights the importance of providing appropriate attention and healthcare to this individuals in clinical practice, as they may be at increased risk of adverse health outcomes that may initially present without symptoms.

## Conclusion

Individuals with NWO showed a worse cardiometabolic profile than those without it. NWO was associated with important anthropometric, body composition, and cardiometabolic variables. Overall, these results corroborate those previously reported. However, further studies on the standardization of cutoff points and methods of assessing body composition in different populations are extremely important. Accurate evaluation and interpretation of body composition, independent of BMI, is crucial in clinical practice to facilitate effective management of medium- and long-term comorbidities associated with excess %BF. Such assessments will help healthcare professionals reduce the risks of negative cardiometabolic complications by providing tailored management strategies for individuals with NWO.
